# Pimavanserin in Alzheimer's Disease Psychosis: Efficacy in Patients with More Pronounced Psychotic Symptoms

**DOI:** 10.14283/jpad.2018.30

**Published:** 2018-08-16

**Authors:** Clive Ballard, J.M. Youakim, B. Coate, S. Stankovic

**Affiliations:** 1 Institute of Health Research, University of Exeter Medical School, EX1 2LU, Exeter, UK; 2 ACADIA Pharmaceuticals Inc., San Diego, CA, USA

**Keywords:** Pimavanserin, Alzheimer's disease, psychosis, severe

## Abstract

**Background:**

Pimavanserin is a 5-HT2A receptor inverse agonist/antagonist and is approved in the United States for the treatment of hallucinations and delusions associated with Parkinson's disease psychosis.

**Objective:**

Evaluate the efficacy of pimavanserin on symptoms of psychosis in patients with Alzheimer's disease (AD).

**Design:**

Randomized, double-blind, placebo-controlled trial

**Setting:**

Nursing home residents

**Participants:**

Patients with AD psychosis

**Interventions:**

Pimavanserin 34 mg or placebo daily for 12 weeks

**Measurements:**

The primary endpoint was mean change from baseline at Week 6 on the Neuropsychiatric Inventory-Nursing Home Version psychosis score (NPI-NH-PS). In the prespecified subgroup analysis, the mean change in NPI-NH-PS and the responder rates among those with baseline NPI-NH-PS ≥12 were evaluated.

**Results:**

Of 181 patients randomized (n=90 pimavanserin; n=91 placebo), 57 had baseline NPI-NH-PS ≥12 (n=27 pimavanserin; n=30 placebo). In this severe subgroup, large treatment effects were observed (delta=-4.43, Cohen's *d*=-0.73, p=0.011), and ≥30% improvement was 88.9% vs. 43.3% (p<0.001) and ≥50% improvement was 77.8% vs. 43.3% (p=0.008) for pimavanserin and placebo, respectively. The rate of adverse events (AEs) in the severe subgroup was similar between treatment groups, and urinary tract infection, fall, and agitation were most frequent. Serious AEs was similar with pimavanserin (17.9%) and placebo (16.7%) with fewer discontinuations due to AEs with pimavanserin (7.1%) compared to placebo (10.0%). Minimal change from baseline occurred for the mean MMSE score over 12 weeks.

**Conclusions:**

Pimavanserin demonstrated significant efficacy in AD psychosis in patients with higher baseline severity of psychotic symptoms (NPI-NH-PS ≥12). Treatment with pimavanserin showed an acceptable tolerability profile.

**W**orldwide, over 40 million people have Alzheimer's disease (AD) or related dementia (1). Neuropsychiatric symptoms such as psychosis including delusions and visual hallucinations occur in 25% to 50% of individuals with AD (2, 3). In addition to occurring in patients with AD, psychosis also occurs in patients with other dementias of a wide variety of etiologies (4). The occurrence of psychotic symptoms in people with AD places a substantial burden on patients with AD, family, and caregivers (1). Patients experiencing psychotic symptoms have more rapid cognitive and functional decline, increased co-morbidity with other neuropsychiatric symptoms including depression and agitation, have higher rates of nursing home admissions, and greater treatment-related mortality (5, 6). An increased severity of psychotic symptoms may be associated with increased disease severity and duration and may be a negative predictor of the overall disease outcomes (7).

Although antipsychotics are commonly used to treat psychosis in patients with dementia and Parkinson's disease (8, 9), randomized, controlled trials of antipsychotics indicate modest efficacy for the treatment of psychosis (10, 11). Meta-analyses of antipsychotic use in patients with AD suggest a small effect size (<0.2) from clinical trials (12, 13), and their use is associated with cognitive decline as well as increased rates of stroke, bronchopneumonia, pulmonary embolism, and mortality (14, 15, 25). However, until recently, no drug was approved for the treatment of the symptoms of psychosis associated with a neurodegenerative disease.

In 2016, based on the results from controlled clinical studies (16, 17) pimavanserin was approved in the United States for the treatment of hallucinations and delusions associated with Parkinson's disease psychosis. Pimavanserin, is a selective 5-hydroxytryptamine (HT)2A receptor inverse agonist/antagonist with substantively lower affinity for the 5-HT2C receptor and negligible affinity for dopaminergic, muscarinic, histaminergic, or adrenergic receptors (18). Results from previous studies suggested that activity at the 5-HT2A receptor could also provide benefits for AD psychosis (19) and formed the basis for a randomized, double-blind, placebo-controlled Phase 2 study where pimavanserin demonstrated significant efficacy in AD patients with psychosis (20). Unlike atypical antipsychotics, pimavanserin did not have a negative impact on cognitive function (20, 21). In addition, no negative effects on motor function were observed with pimavanserin, and the incidence and types of adverse events were comparable with pimavanserin and placebo.

This report describes the efficacy and tolerability of pimavanserin and placebo in a prespecified subgroup of patients with severe psychosis associated with Alzheimer's disease as classified by the cut-off score of ≥12 on the Neuropsychiatric Inventory-Nursing Home Version (NPI-NH) psychosis score (22, 23).

## Methods

Primary results from this Phase 2 study were previously reported (20). In brief, the study was conducted at King's College London in a network of nursing homes across the United Kingdom. An Independent Data Monitoring Ethics Committee provided study oversight. The study was conducted in accordance with the Declaration of Helsinki and the International Council for Harmonisation of Technical Requirements for Pharmaceuticals for Human Use; Good Clinical Practices; and the United States Code of Federal Regulations. Ethics Committee approval was obtained for the study protocol and informed consent form, and patients or their legally authorized representative provided informed consent prior to any study procedures.

### Study Design

This was a randomized, double-blind, placebo-controlled study, with the primary efficacy analysis at the 6-week time point. Study participants continued treatment to 12 weeks with the objective to evaluate overall safety, effects on cognition, and assess maintenance of effect. Patients were randomized equally to pimavanserin 34 mg or placebo once daily, and randomization was stratified by baseline Mini-Mental State Examination (MMSE) (24) in two levels (MMSE <6 and MMSE ≥6) and NPI-NH psychosis score in two levels (hallucinations and delusions <12 and ≥12).

### Patient Selection

Adults ≥50 years of age were eligible if they had possible or probable AD as defined by the National Institute of Neurological and Communicative Disorders and Stroke-Alzheimer's Disease and Related Disorders Association (NINCDS-ADRDA) (25) and satisfying criteria for psychosis associated with Alzheimer's disease (26). Patients were required to have psychotic symptoms including visual and/or auditory hallucinations and/or delusions that developed after the diagnosis of AD was established and must have been a nursing home resident for ≥4 weeks prior to randomization. Further, patients were required to be actively experiencing psychotic symptoms during the month prior to screening that required treatment for psychotic symptoms (26). Patients were required to have a score of ≥4 on either hallucinations (Frequency × Severity) or delusions (Frequency ×Severity) of the NPI-NH psychosis subscale or a total combined score ≥6 (hallucinations + delusions) and have symptoms that required treatment with an antipsychotic medication. Treatment with other antipsychotics or other centrally acting medications was not allowed, and doses of antidepressant and anxiolytic drugs had to remain unchanged during the study. Doses of an acetylcholinesterase inhibitor (and/or memantine) must have been stable for 3 months prior to baseline and during the study. Patients were excluded for a history of significant psychotic disorders prior to or concomitant with the diagnosis of AD, as well as any medical condition that could interfere with the conduct of the study.

### Study Assessments

The NPI-NH psychosis score (hallucinations + delusions) was used to assess psychosis and determine the primary outcome. Behavioral symptoms were assessed using the NPI-NH Total score, as well as the individual behavioral domain scores, and using the Cohen-Mansfield Agitation Inventory-Short Form (CMAI-SF) (27). Cognitive status was evaluated with the MMSE; overall condition was rated with the Alzheimer's Disease Cooperative Study-Clinical Global Impression of Change (ADCS-CGIC) (28); and activities of daily living (ADL) were evaluated using the Alzheimer's Disease Cooperative Study-ADL instrument (ADCSADL) (29). Safety was assessed from adverse events (AEs), physical examinations, clinical laboratory tests, electrocardiograms, and vital signs.

### Statistical Analysis

The primary efficacy outcome was change from baseline to Week 6 for the NPI-NH psychosis score (hallucinations + delusions) for pimavanserin vs. placebo. The focus of the prespecified subgroup analysis was efficacy assessment for baseline NPI-NH psychosis score ≥12 on the primary outcome (NPI-NH psychosis score) and the responder analysis.

For the responder analyses, the reported responder rates were the observed proportions with a response (improvement from baseline) at Week 6, after conservatively imputing any missing values as nonresponders. The treatment groups were compared using a Cochran-Mantel-Haenszel test, stratified by baseline MMSE category (<6 or ≥6). For prespecified efficacy outcomes, the analysis model included fixed effects of baseline MMSE category (<6 or ≥6), treatment (pimavanserin 34 mg or placebo), visit (Days 15, 29, 43, 64, and 85), treatment-by-visit interaction, and a continuous, fixed covariate of baseline score (except for ADCS-CGIC where there is no baseline score). All efficacy analyses used the full analysis set (FAS), which included all randomized participants who received at least one dose of study drug and had both a baseline and at least one post-baseline NPI-NH psychosis score assessment. All efficacy analyses were conducted using 2-sided tests at the 5% significance level. Adverse events were coded using Medical Dictionary for Regulatory Activities (MedDRA) Version 17.0.

## Results

In the overall study population, 181 patients were randomized to pimavanserin (n=90) and placebo (n=91). In the FAS, the group with an NPI-NH psychosis score ≥12 (more severe group) comprised 27 patients randomized to pimavanserin and 30 randomized to placebo. At baseline, demographic and clinical characteristics were generally similar between the overall population and the more severe subgroup (Table 1). However, among patients in the more severe subgroup vs. the overall population, mean NPI-NH total, psychosis, and agitation/aggression scores were higher, and the mean MMSE score was lower, consistent with the overall greater severity of illness (Table 1).

**Table 1:** Baseline demographics and clinical characteristics for the subgroup with NPI-NH psychosis score ≥12 vs. overall population

	**NPI-NH psychosis score ≥12**		**Overall Population**	
	**Pimavanserin (n=27)**	**Placebo (n=30)**	**Pimavanserin (n=87)**	**Placebo (n=91)**
Female, n (%)	22 (81.5)	25 (83.3)	71 (81.6)	73 (80.2)
Age, years^a^	84.9 (7.4)	85.9 (5.3)	85.6 (7.0)	86.1 (6.0)
White, n (%)	23 (85.2)	29 (96.7)	81 (93.1)	89 (97.8)
BMI, kg/m^2^^a^	24.8 (6.7)	22.2 (5.2)	24.1 (5.1)	23.1 (4.6)
Prior Antipsychotic Usage, n (%)	3 (11.1)	4 (13.3)	10 (11.5)	6 (6.6)
Concomitant SSRI, n (%)	8 (29.6)	8 (26.7)	21 (24.1)	20 (22.0)
Concomitant Anti-dementia Medication, n (%)	9 (33.3)	12 (40.0)	33 (37.9)	40 (44.0)
NPI-NH Total Score (10-domain score)^a^	49.0 (15.4)	45.8 (20.6)	33.2 (17.7)	32.9 (19.4)
NPI-NH Psychosis Score^a^	15.3 (4.2)	16.7 (4.5)	9.5 (4.8)	10.0 (5.6)
NPI-NH Agitation/Aggression^a^	7.1 (3.8)	5.9 (4.2)	4·9 (4.0)	4·5 (3.8)
MMSE^a^	8.6 (4.8)	9.2 (5.3)	10·3 (5.4)	9·8 (5.0)
MMSE <6, n (%)	7 (28.0)	5 (19.2)	18 (21.4)	15 (17.6)


^a^ Mean (standard deviation); BMI = body mass index; MMSE = Mini-Mental State Examination; NPI-NH = Neuropsychiatric Inventory - Nursing Home Version; SSRI = selective serotonin reuptake inhibitor.

In the overall population, the adjusted mean change (SE) from baseline to Week 6 (adjusted mean, MMRM analysis) for the NPI-NH psychosis score was −3.76 (0.65) for pimavanserin and −1.93 (0.63) for placebo (delta = −1.84, 95% confidence interval [CI] [−3.64, −0.04] Cohen's *d* = −0.32, p=0.045).

Among patients with baseline NPI-NH psychosis score ≥12, mean baseline scores were 15.3 and 16.7 with pimavanserin and placebo, respectively. The mean change in NPI-NH psychosis score from baseline to Week 6 was −10.15 (95% CI: −12.50, −7.80) for pimavanserin and −5.72 (95% CI: −8.14, −3.30) for placebo (Figure 1A), resulting in a delta of −4.43 (95% CI: −7.81, −1.04) and Cohen's *d* effect size of −0.73 (p=0.011).

**Figure 1A:**
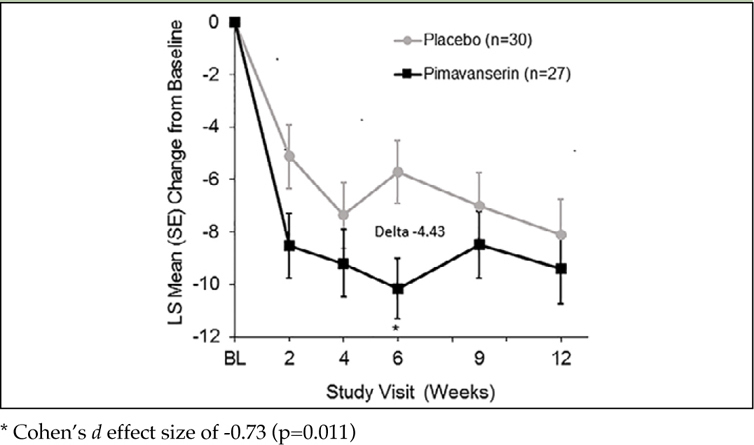
Least square mean change from baseline in NPI-NH psychosis score at each study assessment in participants with baseline NPI-NH psychosis score ≥12 (pimavanserin, n=27; placebo, n=30)

Overall, in the more severe subgroup, 81% of patients had both hallucinations and delusions at baseline. In this subgroup, pimavanserin was superior to placebo in treating both hallucinations and delusions with significant improvements observed at Week 6 for both the NPI-NH hallucinations (p=0.046) and delusions (p=0.034) domain scores (Table 2). Significant differences between pimavanserin and placebo were not observed for other secondary or exploratory outcomes. In the severe subgroup, the change for the NPI-NH psychosis score was significantly (Spearman Correlation=0.4571, p<0.001) correlated with the ADCS-CGIC score at Week 6.

**Table 2:** Secondary and exploratory outcomes for the subgroup with baseline NPI-NH psychosis score ≥12

	**Baseline**		**MMRM LS Mean Change Baseline to Week 6**				
	**Pimavanserin (N=27)**	**Placebo (N=30)**	**Pimavanserin**	**Placebo**	**Delta**	**Effect Size**	**p-value**
NPI-NH Total Score (10-domain score)	49.04	45.83	−22.64	−14.30	−8.34	−0.45	0.114
NPI-NH Delusions	9.89	9.63	−6.39	−4.06	−2.33	−0.61	0.034
NPI-NH Hallucinations	5.41	7.03	−3.65	−1.78	−1.87	−0.57	0.046
NPI-NH Agitation/Aggression	7.11	5.93	−2.38	−2.12	−0.26	−0.06	0.829
NPI-NH Depression/Dysphoria	3.41	3.10	−1.15	−0.94	−0.21	−0.07	0.799
NPI-NH Anxiety	2.81	2.87	−1.17	−0.32	−0.85	−0.25	0.361
NPI-NH Elation/Euphoria	0.78	1.87	−0.70	−0.92	0.22	0.15	0.606
NPI-NH Apathy/Indifference	4.63	3.43	−2.79	−1.65	−1.13	−0.35	0.212
NPI-NH Disinhibition	3.44	2.63	−0.94	−0.87	−0.08	−0.03	0.925
NPI-NH Irritability/Lability	5.67	4.87	−2.11	−0.49	−1.62	−0.43	0.129
NPI-NH Aberrant Motor Behavior	5.89	4.47	−1.19	−1.19	0.01	0.00	0.996
NPI-NH Sleep/Nighttime Behavior	2.81	3.83	−1.41	−0.85	−0.57	−0.18	0.525
NPI-NH Appetite and Eating Changes	2.41	1.33	−0.64	−0.55	−0.09	−0.03	0.908
ADCS-ADL	15.96	14.93	−0.38	−2.90	2.52	0.36	0.192
ADCS-CGIC	−	−	3.44	3.76	−0.31	−0.22	0.425
CMAI-SF Total Score	32.33	32.30	−5.22	−3.97	−1.24	−0.14	0.618
CMAI-SF Aggressive Behavior	8.70	8.63	−1.11	−1.02	−0.09	−0.03	0.919
CMAI-SF Physically Nonaggressive Behavior	11.22	10.20	−0.88	−1.16	0.28	0.07	0.801
CMAI-SF Verbally Agitated Behavior	12.41	13.50	−3.23	−1.87	−1.36	−0.34	0.230

### Responder Analysis

A responder analysis was conducted of the proportion of patients achieving a decrease in the NPI-NH psychosis score of ≥20%, ≥30%, ≥50%, ≥75%, and 100% at Week 6. The proportion with a baseline NPI-NH psychosis score ≥12 achieving a response was significantly (p<0.05) greater with pimavanserin vs. placebo at all increments except for 100% (Figure 1B).

**Figure 1B:**
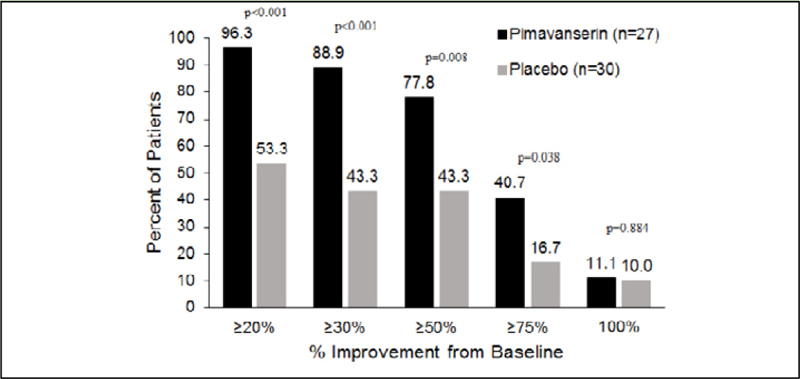
Responder analysis at Week 6 for subgroup with NPI-NH psychosis score ≥12 (pimavanserin, n=27; placebo, n=30)

To enter the study, participants needed to have a score of ≥4 on the either hallucinations or delusions domains of the NPI-NH psychosis scale or an NPI-NH psychosis score ≥6. The baseline score for the severe group was 15.3 and 16.7 for pimavanserin and placebo, respectively (Table 1). At Week 6, 66.7% of pimavanserin patients improved to an NPI-NH psychosis score <6 vs. 32.0% of placebo patients with a treatment difference of 34.7% in favor of pimavanserin. At Week 12, 45.5% of both pimavanserin and placebo-treated patients had an NPINH psychosis score <6.

### Tolerability

**Figure 2:**
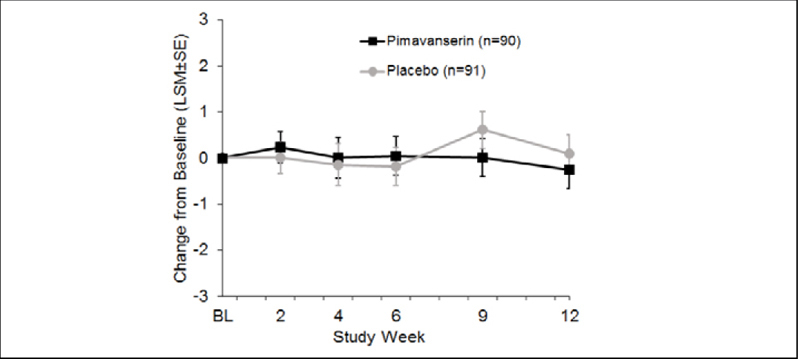
Least square mean change from baseline for MMSE for Safety Population (pimavanserin, n=90; placebo, n=91)

The incidence of adverse events (AEs) was comparable for the more severe (score ≥12) subgroup vs. the overall population (Table 3). In the pimavanserin group, the incidence of aggression was 14.3% in the severe subgroup vs. 10.0% in the overall population, and the incidence of agitation was 17.9% and 21.1% in the severe subgroup and overall population, respectively. The overall incidence of adverse events, serious adverse events, and adverse events causing discontinuation as well as the incidence of all other individual adverse events was similar or lower with pimavanserin in the severe subgroup (Table 3). Minimal change from baseline was observed for the mean MMSE score in either treatment group in the overall study population over 12 weeks of treatment (Figure 2).

**Table 3:** Incidence of adverse events for subgroup with baseline NPI-NH psychosis score ≥12 vs. overall population (Safety Population)

	**Number (%) of Patients**			
	**NPI-NH psychosis score ≥12**		**Overall Population**	
	**Pimavanserin (n=28)**	**Placebo (n=30)**	**Pimavanserin (n=90)**	**Placebo (n=91)**
Summary of Adverse Events				
Any adverse event	27 (96.4)	30 (100)	88 (97.8)	85 (93.4)
Any serious adverse event	5 (17.9)	5 (16.7)	15 (16.7)	10 (11.0)
Any adverse event causing discontinuation	2 (7.1)	3 (10.0)	8 (8.9)	11 (12.1)
Adverse Events ≥5% Overall Pimavanserin Group				
Fall	4 (14.3)	6 (20.0)	21 (23.3)	21 (23.1)
Urinary tract infection	4 (14.3)	9 (30.0)	20 (22.2)	25 (27.5)
Agitation	5 (17.9)	4 (13.3)	19 (21.1)	13 (14.3)
Lower respiratory tract Infection	3 (10.7)	2 (6.7)	13 (14.4)	12 (13.2)
Contusion	4 (14.3)	3 (10.0)	11 (12.2)	14 (15.4)
Aggression	4 (14.3)	2 (6.7)	9 (10.0)	4 (4.4)
Anaemia	2 (7.1)	1 (3.3)	9 (10.0)	8 (8.8)
Blood urea increased	1 (3.6)	3 (10.0)	7 (7.8)	8 (8.8)
Peripheral oedema	1 (3.6)	0	7 (7.8)	2 (2.2)
Cellulitis	1 (3.6)	1 (3.3)	6 (6.7)	3 (3.3)
Anxiety	1 (3.6)	2 (6.7)	5 (5.6)	2 (2.2)
Behavioural and psychiatric symptoms of dementia	1 (3.6)	1 (3.3)	5 (5.6)	2 (2.2)
Blood potassium increased	2 (7.1)	2 (6.7)	5 (5.6)	3 (3.3)

## Discussion

In the overall population of patients with AD psychosis, pimavanserin exhibited efficacy for the primary endpoint, NPI-NH psychosis score, at Week 6 without negative cognitive effects (20). This prespecified analysis was conducted in participants with more severe psychosis at baseline based on an NPI-NH psychosis score ≥12, which represented approximately 30% of the overall study population, a population that is at critical risk for untoward outcomes. While many baseline characteristics were similar between the overall and severe populations, the higher baseline scores for the NPI-NH total, NPI-NH psychosis score, and agitation/aggression domain scores along with lower mean MMSE score supports the hypothesis that the more severe population defined by an NPI-NH psychosis score ≥12 represents a more impaired group of patients with AD psychosis than was represented by the overall population and thus in a greater need for suitable pharmacologic treatment.

For the primary endpoint of mean change in the NPI-NH psychosis score from baseline to Week 6, a notably large Cohen's *d* effect size of −0.73 was observed compared with the effect sizes in the overall population (−0.32). In this vulnerable group of patients, the effect size was more than two-fold greater than reported in the overall study population and more than three-fold greater than the effect size of atypical antipsychotics reported from meta-analyses in populations of patients with dementia-related psychosis (11). Thus, the effect of pimavanserin was markedly greater in the prespecified subgroup with more severe psychotic symptoms. Even more impressive were the results of responder analyses. With 88.9% of patients responding to pimavanserin treatment with at least a 30% reduction in psychotic symptoms and 77.8% responding with at least a 50% reduction, the placebo-adjusted responder rates in this group were in the range of 35%–45%. In addition, a clinically and statistically meaningful reduction in severity was observed with pimavanserin for both hallucination and delusion domains of the NPI-NH. Importantly, these statistically significant findings were observed despite a small sample size in the severe subgroup.

The mean NPI-NH psychosis score in the pimavanserin group maintained the effect through Week 12 in the more severe subgroup but the difference from placebo was not significant due to observed improvement in the placebo group from Weeks 6 and 12. No statistically significant differences were observed for other secondary endpoints although a statistically significant correlation was observed between the NPI-NH psychosis score and the ADCS-CGIC score.

Among patients with AD psychosis, it is reported that an increased occurrence of severe psychosis is associated with an increased presence of delusions and hallucinations as well as symptoms of agitation/aggression (30). Despite an urgent need for effective treatment of these patients, no pharmacological treatment is approved for patients with AD psychosis, in particular, in patients experiencing severe psychotic symptoms. Others have reported an association between the NPI score and the duration and severity of psychosis, which also was associated with the occurrence of delusions and hallucinations (7). In this planned analysis of the severe subgroup from the overall study (20), our results are consistent with these reports, where we found not only a markedly increased NPI-NH total and psychosis score among the severe subgroup at baseline, but also a greater presence of delusions and hallucinations. In addition, baseline factors may have an impact on the magnitude of effect with pimavanserin in the AD population. In the overall study population, prior antipsychotic drug use and increased NPI-NH agitation/aggression scores at baseline were associated with greater effect sizes with pimavanserin that were significant for prior antipsychotic use (p=0.037 and p=0.001, respectively).

Limitations of this analysis are the small number of patients included in the severe subgroup and the secondary nature of this subgroup analysis. However, patients included in this prespecified analysis were from a prospective, randomized, placebo-controlled study, and a significant difference was observed for the primary outcome, the NPI-NH psychosis score in the overall population. The findings of an association between dementia-related psychosis and neuropsychiatric complaints are consistent with previous reports in patients with dementia and suggest that this severe population is at greater risk for adverse outcomes that require effective treatments (3, 6, 8).

The robust efficacy of pimavanserin in patients with more severe psychotic symptoms is relevant to the therapeutic benefits of pimavanserin in patient populations with AD and psychosis. These results extend and confirm the results from the primary analysis in the overall population (20) and the results from the subgroup analysis of patients with mild dementia in the PDP study with pimavanserin (16). These findings coupled with the results from other studies of pimavanserin suggest a potential role for pimavanserin in treating psychosis in patients across a range of neuropsychiatric conditions.

*Acknowmedgement:* The authors acknowledge the editorial assistance of Richard S. Perry, PharmD in the preparation of this manuscript, which was supported by ACADIA Pharmaceuticals Inc., San Diego, California.

*Funding:* This study was funded by ACADIA Pharmaceuticals Inc., San Diego, California. All authors as well as the sponsor were involved in the design and conduct of the study; the collection, analysis, and interpretation of data; in the preparation of the manuscript; and in the review or approval of the manuscript.

*Disclosures:* Dr. Ballard has received grants and personal fees from ACADIA and Lundbeck, personal fees from Heptares, Roche, Lilly, Otsuka, Orion, GlaxoSmithKline, and Pfizer. JY, BC, and SS, are employees and may be stockholders in ACADIA Pharmaceuticals Inc.

*Ethical standard:* The study adheres to the Declaration of Helsinki human protection guidelines and was reviewed by ethical standards boards for all participating sites.
